# 

**Published:** 1882-07

**Authors:** 


					﻿wTR5le Number,
CCXXXXII.
JULY, 1882.
Number 12,
Volume XXI.
THE
BUFFALO
Medical and Surgical Journal.
EDITORS:
THO9. I.OTHROP, M. D„
F*. W. VAN PEYMA, M. I).,
A. R. DAVIDSON, M.
LVCIEN HOWE, M.
HERMAN MVNTER, M. D.
□3 TT ZEr'ZE1-A. Xi O z
Published by the Medical Journal Association.
Read the Notice of this Journal among the Advertisements.
Entered at the Post-Office at Buffalo, N. Y., as Second-Class Mail Matter.
				

